# Using linked electronic health records to report healthcare-associated infections

**DOI:** 10.1371/journal.pone.0206860

**Published:** 2018-11-07

**Authors:** T. Phuong Quan, Russell Hope, Tiphanie Clarke, Ruth Moroney, Lisa Butcher, Peter Knight, Derrick Crook, Susan Hopkins, Timothy E. A. Peto, Alan P. Johnson, A. Sarah Walker

**Affiliations:** 1 The National Institute for Health Research (NIHR) Health Protection Research Unit in Healthcare Associated Infections and Antimicrobial Resistance at University of Oxford, Oxford, United Kingdom; 2 Nuffield Department of Medicine, University of Oxford, Oxford, United Kingdom; 3 National Infection Service, Public Health England, Colindale, London, United Kingdom; 4 Oxford University Hospitals NHS Foundation Trust, Oxford, United Kingdom; 5 NIHR Biomedical Research Centre, Oxford, United Kingdom; University of New South Wales, AUSTRALIA

## Abstract

**Background:**

Reporting of strategic healthcare-associated infections (HCAIs) to Public Health England is mandatory for all acute hospital trusts in England, via a web-based HCAI Data Capture System (HCAI-DCS).

**Aim:**

Investigate the feasibility of automating the current, manual, HCAI reporting using linked electronic health records (linked-EHR), and assess its level of accuracy.

**Methods:**

All data previously submitted through the HCAI-DCS by the Oxford University Hospitals infection control (IC) team for methicillin-resistant and methicillin-susceptible *Staphylococcus aureus* (MRSA, MSSA), *Clostridium difficile*, and *Escherichia coli*, through March 2017 were downloaded and compared to outputs created from linked-EHR, with detailed comparisons between 2013–2017.

**Findings:**

Total MRSA, MSSA, *E*. *coli* and *C*. *difficile* cases entered by the IC team vs linked-EHR were 428 vs 432, 795 vs 816, 2454 vs 2450 and 3365 vs 3393 respectively. From 2013–2017, most discrepancies (32/37 (86%)) were likely due to IC recording errors. Patient and specimen identifiers were completed for >98% of cases by both methods, with very high agreement (>97%). Fields relating to the patient at the time the specimen was taken were complete to a similarly high level (>99% IC, >97% linked-EHR), and agreement was fairly good (>80%) except for the main and treatment specialties (57% and 54% respectively) and the patient category (55%). Optional, organism-specific data-fields were less complete, by both methods. Where comparisons were possible, agreement was reasonably high (mostly 70–90%).

**Conclusion:**

Basic factual information, such as demographic data, is almost-certainly better automated, and many other data fields can potentially be populated successfully from linked-EHR. Manual data collection is time-consuming and inefficient; automated electronic data collection would leave healthcare professionals free to focus on clinical rather than administrative work.

## Introduction

Effective surveillance of healthcare-associated infections (HCAIs) requires demographic, clinical and epidemiological information to be collected on a case-by-case basis, which can result in vast amounts of effort for infection control (IC) teams. In England, the reporting of certain strategic HCAIs is mandatory[1] for all acute hospital trusts as well as the independent sector.

When mandatory HCAI surveillance began in 2001[2], only quarterly laboratory data on numbers of blood cultures taken and specific positive isolations, namely methicillin-resistant *Staphylococcus aureus* (MRSA), were requested. By 2011, hospitals were required to submit individual patient-level data on a monthly basis, further covering methicillin-susceptible *S*. *aureus* (MSSA) and *Escherichia coli* blood culture isolates as well as cases of *Clostridium difficile* infection. Reporting of bloodstream infections due to *Klebsiella* spp. and *Pseudomonas aeruginosa* was made mandatory on 1^st^ April 2017, and other infectious organisms will also likely become important for surveillance in the future[3], adding further to the burden of reporting by trusts. When data input is undertaken manually, it is not only time-consuming for frontline clinical staff but can also easily be subject to transcription and typographical errors.

The implementation of electronic surveillance software for healthcare-associated infections has been found to be feasible in many settings, though uptake has been slow, and most studies on its impact have been based in the USA[4, 5]. At the Oxford University Hospitals NHS Foundation Trust (OUH), England, we have implemented a near-real-time linkage of multiple hospital datasets[6] (consisting of data generated during routine interactions between patients and the healthcare system), with the purpose of supporting infection surveillance, audit and decision-making. We used this linked database of routinely collected electronic health records (linked-EHR), which includes admissions and microbiological data, to investigate whether it would be possible to automate the current manual reporting by laboratories or IC teams, and to what level of accuracy.

## Methods

Data for mandatory surveillance are submitted to Public Health England (PHE) via a web-based system called the HCAI Data Capture System (HCAI-DCS). We worked alongside the OUH IC team to understand the information required for each HCAI-DCS data field, and downloaded all data submitted in previous years (from 1^st^ April 2005 for MRSA, 1^st^ April 2007 for *C*. *difficile*, 1^st^ January 2011 for MSSA and 1^st^ June 2011 for *E*. *coli*, all to 31^st^ March 2017 inclusive). Completion of certain data fields is compulsory, such as the specimen date and patient identifiers, while others are optional, such as patient risk factors and prior healthcare interactions. Individual infection episodes are de-duplicated within 14-days for bacteraemia cases or 28-days for cases of C. difficile infection. Quarterly-aggregated numbers of laboratory tests are also entered into the HCAI-DCS, (often directly by the laboratory instead of by the IC teams), and these were also downloaded.

We identified the data fields which could potentially be extracted from the linked-EHR, and wrote databases queries based on generic rules, in order to generate these fields automatically for each case (see supplementary material). We included inpatient admissions (including diagnostic codes), outpatient appointments, and microbiology data only.

We assessed accuracy firstly by comparing overall numbers of infections found (after episode de-duplication) across the entire period each organism was subject to mandatory surveillance, and secondly by comparing the details entered for individual cases from April 2013–March 2017 to best represent current practice, linking on specimen number and date. In October 2015 a new version of the HCAI-DCS was released and so we also compared data quality between the two versions. We quantified the completeness of each data field across individual cases, and when they contained a value from both IC teams and linked-EHR, whether this was an exact match or not, (for each field, and for each related group of fields). When comparing prior healthcare interactions within the same trust, agreement was simply based on whether or not *any* prior interaction had been recorded. We investigated possible explanations for discrepancies but did not attempt to verify which value was actually correct (e.g. by retrieving medical notes).

Ethics: this study was conducted as a quality improvement project within the NHS and therefore did not require Research Ethics Committee review. The study was approved by Oxford University Hospitals NHS Foundation Trust.

## Results

**Fig 1:**
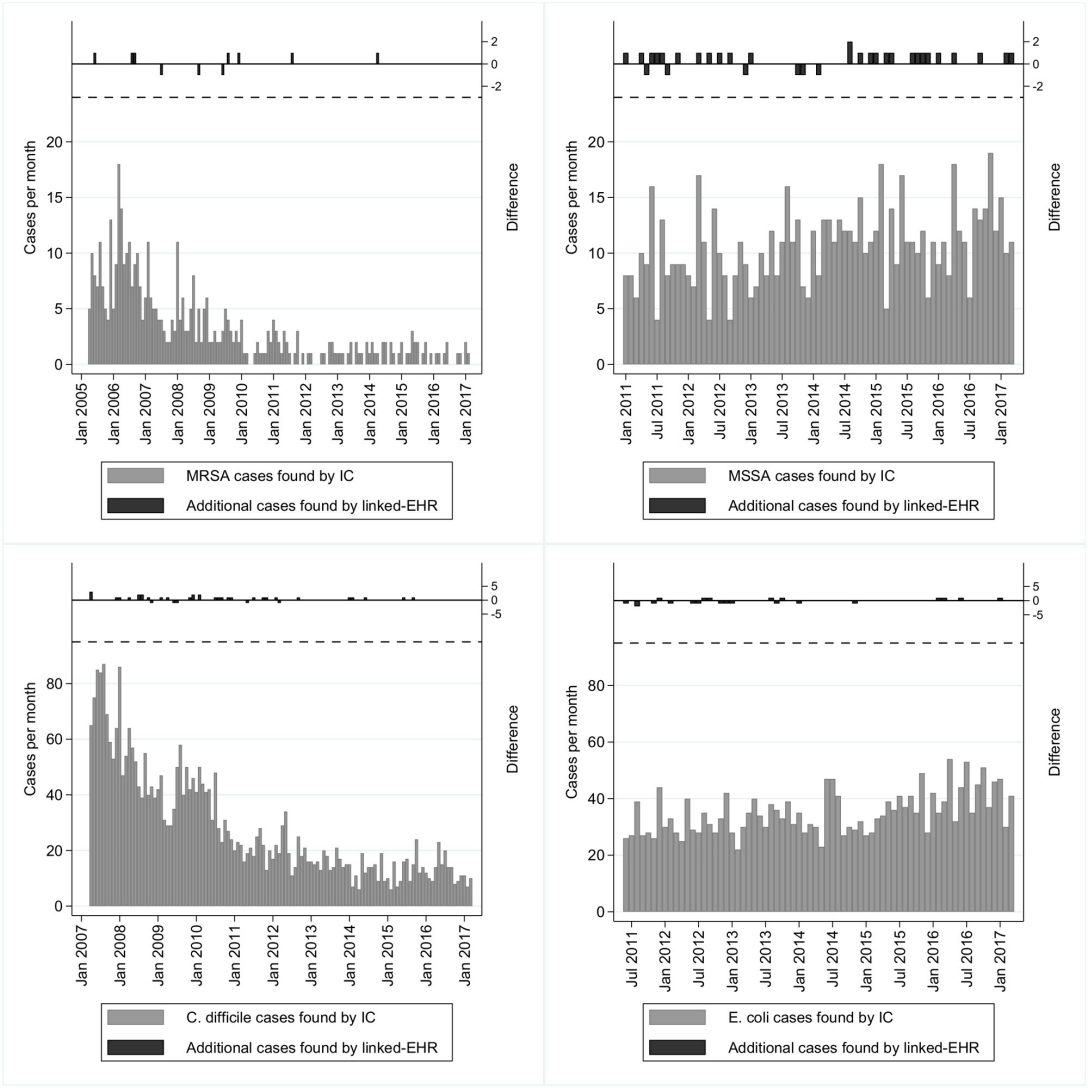
Total numbers of individual cases recorded each month by the infection control (IC) team versus the number found in the linked-EHR. The lower section of each graph shows the total number of cases entered by the IC team. The upper section shows the difference compared to linked-EHR: a positive number indicates more cases found by linked-EHR, a negative number indicates more cases found by IC.

The total numbers of (de-duplicated) MRSA, MSSA, *E*. *coli* and *C*. *difficile* cases entered into HCAI-DCS by the OUH IC team, over the relevant reporting periods (from 1^st^ April 2005 for MRSA, 1^st^ April 2007 for *C*. *difficile*, 1^st^ January 2011 for MSSA and 1^st^ June 2011 for *E*. *coli*, all to March 2017 inclusive) were 428, 795, 2454, and 3365 respectively; the total numbers in linked-EHR were very similar overall at 432 (+4 (0.9%)), 816 (+21 (2.6%)), 2450 (-4 (0.2%)), and 3393 (+28 (0.8%)) (Fig 1).

Between April 2013 and March 2017 inclusive, 37 cases were identified by linked-EHR but not by IC and 7 cases were identified by IC and not by linked-EHR (Table 1). In 13/37 (35%) discrepancies, an error appeared to have been made by the IC team, (e.g. incorrectly calculating the episode de-duplication window), and in five (14%) discrepancies an error was made by linked-EHR, (e.g. not reading the free text that indicated the sample had been mislabelled). In 19 cases (51%), an infection was identified by linked-EHR but not by IC and had no obvious reason to be missing, although the most plausible explanation is that they were accidentally missed by the IC team.

**Table 1:** Cases reported by the infection control (IC) team that could not be matched to a case extracted by linked-EHR, and vice versa, from April 2013-March 2017, with the most likely explanation for the discrepancy.

Type of discrepancy (n) Total n = 37	Likely error by IC team (n) Total n = 13	Likely error by linked-EHR (n) Total n = 5	No obvious cause of discrepancy (n) Total n = 19
MRSA only found by linked-EHR (1)	Child under 2 years* (1)	-	-
MRSA only found by IC (0)	-	-	-
MSSA only found by linked-EHR (18)	Child under 2 years* (1) Potentially de-duplicated based on an MRSA bacteraemia 2 days earlier in same patient (1) Potentially de-duplicated based on incorrect NHS number entered (1)	Pleural fluid sample tested as blood culture. Previous positive BLC 15 days before (1)	No explanation found† (14)
MSSA only found by IC (4)	Post-mortem specimen (4)	-	-
C. difficile only found by linked-EHR (5)	Episode de-duplication window miscalculated, i.e. a new case 29 days after a previous case was not entered (2)	Free text on the microbiology database indicated a labelling error with the sample or that the test was ordered in error, and thus that the result should be ignored (2)	No explanation found† (1)
C. difficile only found by IC (0)	-	-	-
E. coli only found by linked-EHR (6)	-	Linkage algorithm incorrectly identified a sample as belonging to a new patient instead of as a duplicate of a previous case (1) Free text on the microbiology database indicated that the clinical description on the form did not match the patient and so the result should be treated with caution (1)	No explanation found† (4)
E. coli only found by IC (3)	Post-mortem specimen (2) Episode de-duplication window miscalculated (1)	-	-

* this is an exclusion criterion for *C*. *difficile* cases but not *S*. *aureus*

† for all these 19 cases we identified a positive culture from the microbiology laboratory in linked-EHR without a corresponding record on HCAI-DCS. The most plausible explanation is that these were accidentally missed by the IC team

**Fig 2:**
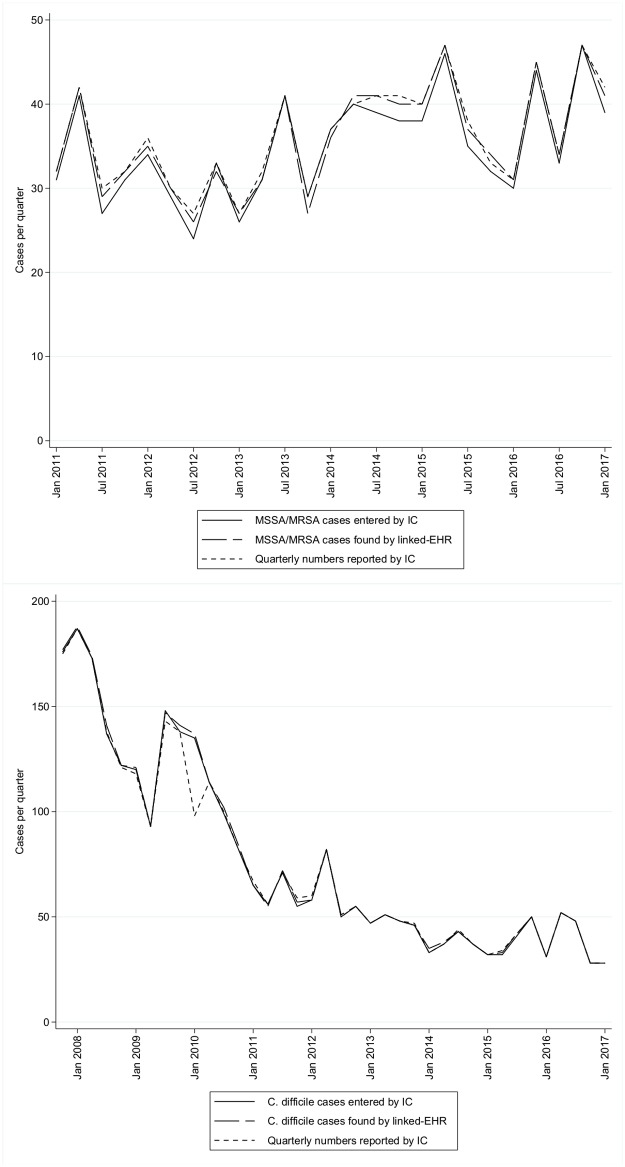
Quarterly-aggregated numbers of infections identified. This compares the quarterly-aggregated numbers reported by the infection control (IC) team versus the number of individual cases entered in that quarter, versus the number found in the linked-her.

Quarterly-aggregated summary numbers are entered separately into HCAI-DCS by the IC team, and are only collected for MRSA/MSSA combined, as well as for *C*. *difficile*. These numbers matched the numbers from linked-EHR very closely except for a single *C*. *difficile* entry, which was likely a typographical error (Fig 2).

Table 2 shows the level of completeness and agreement (when both methods produced a value) of data fields that are common across all four organisms. Patient and specimen identifiers were completed for >99% cases by both methods, and to a very high level of agreement (>98%). Discrepancies appeared to be predominantly caused by transcription errors made by the IC team. Fields relating to when the specimen was taken were complete to a similarly high level (>99% by IC, >97% by linked-EHR), and agreement was fairly good (>80%) except for the main and treatment specialties (57% and 54% respectively) and the patient category (55%). The most common discrepancy (28% of discrepancies) in main specialty was where linked-EHR specified “Geriatric medicine” but IC specified “Acute general medicine”, and the most common discrepancy (47% of discrepancies) in treatment specialty was where linked-EHR specified “Accident and Emergency” but IC specified “Acute general medicine”. Most discrepancies (59%) in patient category occurred where linked-EHR specified “A&E only” but IC specified “Emergency assessment”. The fact that the A&E category was less commonly used by the IC team suggests that they may have been recording the dominant specialty the patient was under for that admission when they should have been recording the specialty at the time the sample was taken, as per HCAI-DCS guidance.

**Table 2:** The completeness and agreement of data fields common across all four organisms, based on 3005 individual cases from April 2013-March 2017.

Field (Total cases = 3005)	Required (R) or optional (O) field	Completeness by IC (%)	Completeness by linked-EHR (%)	Agreement where both complete (%)
***Patient identifiers***:		***99*.*8***	***99*.*8***	***98*.*5***
NHS Number	R	99.1	99.2	99.8
Hospital number	O	99.8	99.8	98.9
Date of birth	R	100.0	100.0	98.6
Sex	R	99.8	100.0	99.7
Forename	R	99.9	100.0	96.8
Surname	R	100.0	100.0	97.2
***Specimen identifiers***:		***100*.*0***	***100*.*0***	***99*.*0***
Specimen date	R	100.0	100.0	99.1
Type of specimen date (i.e. date taken or date received)		100.0	100.0	99.9
Specimen number	O	100.0	100.0	97.4
Laboratory where specimen was processed	O	100.0	100.0	99.7
***Information about the patient when specimen was taken***:		***99*.*3***	***97*.*6***	***83*.*8***
Location specimen taken (e.g. acute hospital, GP)	R	100.0	99.4	95.9
Hospital site	R*	100.0	99.7	97.3
Patient category (e.g. inpatient, outpatient, A&E only)	R	99.9	100.0	54.5
Date admitted (if admitted)	R*	100.0	99.4	93.0
Admission method (e.g. emergency, waiting list)	R*	99.9	99.4	90.6
Main specialty (of consultant)	R*	100.0	89.6	56.9
Treatment specialty	R*	99.7	96.0	54.0
Augmented care	R*	99.8	100.0	96.6
Provenance of patient (e.g. home, nursing home)	O	98.9	90.4	81.1
Episode category (e.g. new infection, repeat/relapse)†	O	98.4	97.5	96.4
On dialysis (e.g. acute renal failure, established renal failure, not on dialysis, unknown)	R	98.6	100.0	97.6
Admitted any time this episode (Y/N)	O	96.2	100.0	90.1

* Required if triggered by earlier answer

† This field was originally only available for *C*. *difficile* and *E*. *coli*, but was expanded to include MRSA/MSSA in October 2014

Table 3 shows the completeness and agreement of all other, optional, organism-specific data fields. These were less complete than for the common fields, by both methods. The IC team generally completed fields more thoroughly for *S*. *aureus* (overall 81% complete) and *E*. *coli* (overall 74% complete) than for *C*. *difficile* (overall 48% complete). Of note, no data were entered at all for the renal section, because the IC team were unaware before this study that there were additional actions that needed to be taken for those cases. For linked-EHR, completeness was either 100% or 0%, based on whether or not an algorithm could be constructed from the available electronic data; no information could be extracted for fields which related to the root causes or treatment of the infection (as opposed to those relating to the hospital stay as a whole). Where comparisons could be made, agreement was reasonably high (mostly 70–90%).

**Table 3:** Completeness and agreement of organism-specific data fields, and for patients on dialysis. Completion of these sections is optional.

Field (all optional)	Completeness by IC (%)	Completeness by linked-EHR (%)	Agreement where both complete (%)
***MRSA/MSSA (595 cases)***:	**80.5**	**38.0**	**83.0**
Risk factors	75.0	73.0	85.2
Assisted ventilation–past 7 days	74.5	100.0	92.6
Assisted ventilation—current	74.6	100.0	93.8
Central IV device	77.5	0.0	-
Diabetic	79.3	100.0	91.9
IV drug user	75.0	0.0	-
Immunosuppressed	77.8	100.0	81.5
Liver disease	74.3	100.0	93.1
Peripheral IV device	78.5	0.0	-
Prior *S*. *aureus* history	89.7	100.0	76.0
If yes when	51.4	100.0	57.9
Prosthesis	75.7	100.0	75.9
Surgical wound	77.5	100.0	87.9
Urinary catheter	77.7	100.0	80.1
Other	37.7	0.0	-
Treatment (e.g. antibiotic given, wound drained, catheter removed)	100.0	0.0	-
Source of bacteraemia & associated infections	79.7	0.0	-
Source of bacteraemia	70.4	0.0	-
Certainty	72.9	0.0	-
Associated clinical infection	80.7	0.0	-
Certainty	66.5	0.0	-
Specialty where infection thought to have been acquired (if inpatient)			
Augmented care specialty	97.8	0.0	-
Treatment specialty	80.8	0.0	-
Date (in specialty) from	84.0	0.0	-
Date (in specialty) to	90.3	0.0	-
Prior healthcare interactions in this trust	68.6	100.0	61.2*
Prior healthcare interactions in other trust	68.6	0.0	-
***C*. *difficile (637 cases)***:	**48.3**	**5.4**	**39.7**
Best estimate of date of onset of diarrhoea	72.1	0.0	-
Antimicrobial usage			
Was patient on antimicrobials when specimen was taken	72.5	0.0	-
Was patient on any other antimicrobials in preceding 7 days	70.8	0.0	-
Prior healthcare interactions in this trust	68.4	100.0	39.7*
Prior healthcare interactions in other trust	47.1	0.0	-
Specialty where infection thought to have been acquired (if inpatient)			
Augmented care specialty	55.7	0.0	-
Treatment specialty	44.9	0.0	-
Date (in specialty) from	46.4	0.0	-
Date (in specialty) to	44.3	0.0	-
Discharge date	16.1	0.0	-
Discharge type	53.4	0.0	-
Total number of beds (in whole ward or unit)	47.5	0.0	-
Ward type (e.g. single room, 4-bedded bay)	53.3	0.0	-
Reference laboratory result			-
Was the specimen sent for typing	71.1	0.0	-
Date sent	0.1	0.0	-
Specimen category	0.1	0.0	-
***E*. *coli (1773 cases)***:	**73.6**	**0.0**	-
Most likely primary focus	82.6	0.0	-
Factors directly predisposing to this episode	90.9	0.0	-
Urinary catheterisation	62.6	0.0	-
Vascular access device	59.2	0.0	-
Other invasive/indwelling device	58.6	0.0	-
Surgical or other invasive procedure	62.1	0.0	-
Neutropenia	64.4	0.0	-
Wound/ulcer	59.5	0.0	-
Other factors	66.8	0.0	-
Is this episode likely to be HCAI	97.5	0.0	-
If yes, where from (e.g. current admission, previous acute admission)	84.2	0.0	-
***Renal***† ***(73 cases)***:	**0.0**	**32.7**	-
Usual provider of renal care:			
Mother unit (hub)	0.0	82.2	-
Satellite unit	0.0	0.0	-
Other & non-UK etc	0.0	0.0	-
Dialysis details:			
Modality	0.0	82.2	-
Type of access being used	0.0	64.4	-
Catheter last 28/7	0.0	0.0	-
If Yes, what type	0.0	0.0	-

* For prior healthcare interactions within the same trust, agreement was simply based on whether or not *any* prior interaction had been recorded

† The renal section should actually be entered by the renal teams rather than the IC teams but still require the IC team to ‘share’ the record first, an action they weren’t aware of before this study

After the new version of the HCAI-DCS web-application was released, the completeness of optional data fields input by the IC team (based on the 30 months pre- versus 18 months post-release) increased for *S*. *aureus* (from 73% to 94%) and *E*. *coli* (from 66% to 85%) but surprisingly decreased for *C*. *difficile* (from 52% to 41%). The subcategories with the most notable increases were *S*. *aureus* risk factors (from 64% to 97%) and prior healthcare interactions (from 51% to 99% for *S*. *aureus* and 49% to 74% for *C*. *difficile*). The main source of the decrease in *C*. *difficile* completion was in fields related to the specialty where the infection was thought to have been acquired (from 58% to 28%). The completeness of other subcategories did not appear to change substantially.

## Discussion

By comparing the completeness and concordance of data manually collated by IC teams versus that automatically extracted from linked-EHR, we found that basic factual information, such as demographic data, is almost-certainly better automated. Perhaps more surprisingly, even certain optional fields, such as “current liver disease”, were relatively easy to identify from linked-EHR and were highly concordant. Automatic extraction could potentially be improved by further tuning of the algorithm as well as by incorporating additional data sources such as electronic prescribing. Fields relating to the root causes of the infection are unlikely to ever be automatable; however, if it is only these types of fields that the IC team need to focus on it would still reduce their work considerably. The release of a new, more user-friendly HCAI-DCS web-application generally increased the completeness of data entered by IC.

The main limitation of this study is that it was conducted at a single site, with its own particular processes around IC reporting. All trusts should have dedicated IC teams, but are likely to have different levels of completeness and accuracy in their IC reporting, which may also vary over time; however, the types of errors we identified would plausibly occur at other sites as well. The study was also not conducted in real-time, with the possibility that some data may have been updated or corrected in hospital systems after the HCAI-DCS reporting window closed, though the easiest gains (i.e. typographical and de-duplication errors) are unlikely to be affected by this. A further limitation is that we did not conduct a clinical notes review to identify which method was actually more accurate in the case of discrepancies. However, we did identify situations in which guidance had not been consistently followed by IC teams, whereas linked-EHR provided consistent attributions based on the underlying source data.

A large amount of data is already being collected and transferred electronically within the NHS; for instance all admissions data is sent to the Secondary Uses Service[7] at NHS Digital to form Hospital Episode Statistics (HES). Laboratories serving most trusts also electronically transfer microbiological test results to the voluntary surveillance database (Second Generation Surveillance System (SGSS)) at PHE, with latest known case ascertainment rates of 75%[8], 69%, and 93%[9] for MRSA, MSSA and *E*. *coli* respectively in 2016, and 88%[10] for *C*. *difficile* in 2014, compared to the mandatory reporting scheme. The missing cases are most likely due to microbiology laboratories representing entire trusts not participating in the voluntary scheme (as opposed to incomplete reporting within laboratories) as well as potential differences in case definitions.

Since these data are already being collected electronically on a national scale, the automation assessed in this study could feasibly be conducted at other trusts as well (and already has in some[11]). However, this will require significant investment for trusts without existing linked systems, although the increasing use of electronic sample submission and near universal use of NHS numbers makes this more straightforward than it would have been historically. Mapping data fields from our EHR system to the HCAI-DCS was not a trivial exercise and so the ability to build on previous efforts such as ours, (as detailed in the supplementary material), should make it easier for other trusts to make their own attempts. However, each new in-house system should still undergo its own validation exercises.

A further feature of the updated HCAI-DCS is the ability to upload files containing the common data fields for multiple cases simultaneously, rather than having to fill in the online forms individually. Nineteen trusts are already using this feature, with at least two (Imperial College Healthcare[11] and Royal Free London (S. Hopkins, personal communication)) including electronically linked admissions data in their submissions as well as populating the microbiology results. The fact that many trusts are already using the feature means there is certainly some appetite for automation across the country.

Alternatively, if the automated extraction could be centralised at PHE by linking HES and SGSS data, then not only would this mean no greater work for individual trusts, but it might also mean more consistent data being collected, since algorithms would be applied consistently across all trusts and would not rely on individual interpretation of the guidance nor on differing levels of available resources (although trusts which use external laboratories may need additional support to encourage SGSS participation and ensure data-feed accuracy by their laboratory partner). Importantly, it would also make it very easy to update or expand the list of infections covered at will, without increasing trusts’ already-heavy workload. Furthermore, it would enable information such as interactions with other trusts to be automatically calculated. Although this is completed by local IC teams, their knowledge may be incomplete, and local linked-EHR is only able to assess prior exposures within-trust. This may be much less accurate in urban areas with multiple trusts (e.g. London) than Oxfordshire. Historically, infection numbers have contributed to financial penalties, so a system for reconciling centralised linked-EHR surveillance and local data would be needed, at least until enough confidence had been gained that the algorithms were correct, as well as to enable correction of any errors in automated feeds.

An important question is where the trade-off between completeness and accuracy should lie, particularly for the optional data fields. A human can investigate non-straightforward cases more thoroughly than a generic algorithm, particularly when not all relevant information is available electronically. However, as seen in our study, humans may only have time to collect optional data for a subset of cases whilst an algorithm would collect it consistently for all cases. Another option would be to focus on retrospectively collecting labour-intensive data on a representative sample of cases, rather than trying to collect it for all cases. And while it is clearly desirable to have this additional information, if it is not done well or completely enough to make informed decisions with, there is probably little value in collecting it at all. Another potential trade-off is in timeliness, as the window for reporting new infections is currently very strict, i.e. the 15th day after the month in which the infection was identified (although the optional data fields can be populated at a later date). SGSS data is submitted reasonably promptly but HES data (needed at a minimum to determine hospital-onset cases) is only released after data cleaning by NHS Digital, and so would not be available for the 15^th^. Again though, any delays may be outweighed by the benefits gained in data consistency.

## Conclusions

More and more data are being recorded electronically, and greater importance is being placed on using this data effectively[11]. Furthermore, manual data collection is time-consuming, inefficient, and has intrinsic opportunity costs. While this study focussed specifically on the reporting requirements for healthcare-associated infections in England, the principles are applicable to any large-scale reporting schemes as well as to any IT systems. Where there are limitations in the performance of algorithms for more nuanced questions, (e.g. ascribing the root cause of an infection), a hybrid system combining automatic generation of reports with subsequent manual review could potentially be implemented. Ultimately, transferring tasks which could be done more effectively and consistently by computer would leave healthcare professionals free to do more valuable work managing infections and potential outbreaks rather than doing data entry.

## Supporting information

S1 Table Algorithms used to create the linked-EHR extract for fields common to all organisms. (DOCX)

S2 Table Algorithms used to create the linked-EHR extract for organism-specific fields. (DOCX)

S1 File ICD-10 diagnosis codes used in algorithm for MRSA/MSSA Surgical wound risk factor. (XLSX)
