# Supplementary material for: Using linked electronic health records to report healthcare-associated infections
Source: PLoS One. 2018 Nov 7;13(11):e0206860. doi: 10.1371/journal.pone.0206860 (PMC6221334; doi:10.1371/journal.pone.0206860)
Supplement: S1 Table — (DOCX) [file pone.0206860.s001.docx]

**S1 Table. Algorithms used to create the linked-EHR extract for fields common to all organisms**

| **Common Fields** | **Algorithm** |
| --- | --- |
| ***Information about the patient when specimen was taken:*** |  |
| Location specimen taken (e.g. acute hospital, GP) | Based on SpecimenCollectionLocation (from lab system) |
| Hospital site | Based on SiteCode (from PAS system) if inpatient (i.e. specimen date between episode start and end date inclusive) or outpatient (i.e. specimendate same as appointmentdate) |
| Patient category (e.g. inpatient, outpatient, A&E only) | Based on SpecimenCollectionLocation (from lab system), and PatientClassificationCode (from PAS system) if inpatient at time of sample (i.e. specimen date between episode start and end date inclusive) |
| Date admitted (if admitted) | AdmissionDate (from PAS system) if inpatient at time of sample (i.e. specimen date between episode start and end date inclusive) |
| Admission method (e.g. emergency, waiting list) | AdmissionMethod (from PAS system) if inpatient at time of sample (i.e. specimen date between episode start and end date inclusive) |
| Main specialty (of consultant) | MainSpecialty (from PAS system) if inpatient (i.e. specimen date between episode start and end date inclusive) or outpatient (i.e. specimendate same as appointmentdate) |
| Treatment specialty | TreatmentSpecialty (from PAS system) if inpatient (i.e. specimen date between episode start and end date inclusive) or outpatient (i.e. specimendate same as appointmentdate) |
| Augmented care | Based on SpecimenCollectionLocation (from lab system) |
| Provenance of patient (e.g. home, nursing home) | Based on AdmissionSourceCode (from PAS system) if inpatient (i.e. specimen date between episode start and end date inclusive), else ‘Home’ if SpecimenCollectionLocation (from lab system) is GP or outpatient |
| Episode category (e.g. new infection, repeat/relapse)† | If a previous positive sample found in lab system within 60 days of specimen date then ‘Unknown’, else ‘New infection’ |
| On dialysis (e.g. acute renal failure, established renal failure, not on dialysis, unknown) | For any inpatient episodes (including current episode) within 60 days of specimen date:  ‘Acute RF’ if any ICD10 diagnosis code in N17, N17.0, N17.1, N17.2, N17.8, N17.9 or N19  ‘Established RF’ if any ICD10 diagnosis code in N18, N18.5, N18.9, Z49, Z49.0, Z49.1, Z49.2 or Z992  Else ‘No’ |
| Admitted any time this episode (Y/N) | ‘Y’ if inpatient at time of sample (i.e. specimen date between episode start and end date inclusive) |
