# Supplementary material for: Using linked electronic health records to report healthcare-associated infections
Source: PLoS One. 2018 Nov 7;13(11):e0206860. doi: 10.1371/journal.pone.0206860 (PMC6221334; doi:10.1371/journal.pone.0206860)
Supplement: S2 Table — (DOCX) [file pone.0206860.s002.docx]

**S2 Table. Algorithms used to create the linked-EHR extract for organism-specific fields**

| **Organism-specific Fields** | **Algorithm (* denotes a wildcard character)** |
| --- | --- |
| ***MRSA/MSSA:*** |  |
| Risk factors |  |
| Assisted ventilation – past 7 days | ‘Y’ if OPCS procedure code in E85, E85.1, E85.2, E85.3, E85.4, E85.5, E85.8 or E85.9, and procedure date within 7 days of specimen date, (for any inpatient episodes (including current episode) within 7 days of specimen date) |
| Assisted ventilation – current | ‘Y’ if OPCS procedure code in E85, E85.1, E85.2, E85.3, E85.4, E85.5, E85.8 or E85.9, and procedure date same as specimen date, (for any inpatient episodes (including current episode) within 7 days of specimen date) |
| Central IV device | - |
| Diabetic | ‘Y’ if any ICD10 diagnosis code in E10.*, E11.*, E13.*, E14.*, R73, R73.0, R73.9, R81 or R82.4, (for any inpatient episodes (including current episode) within 60 days of specimen date) |
| IV drug user | - |
| Immunosuppressed | ‘Y’ if any ICD10 diagnosis code in D80.*, D81.*, D82.*, D83.*, D84.* or D89.*, (for any inpatient episodes (including current episode) within 60 days of specimen date) |
| Liver disease | ‘Y’ if any ICD10 diagnosis code in A06.4, K70.*, K71.*, K72.*, K74.*, K75.*, K76.*, K77.*, R16.*, R17, R18, R74.*, or R94.5, (for any inpatient episodes (including current episode) within 60 days of specimen date) |
| Peripheral IV device | - |
| Prior *S. aureus* history | Based on lab test code of previous sample:  ‘Bacteraemia’ if blood culture  ‘Colonised’ if screening sample  ‘Infection’ if neither of above |
| If yes when | Based on days between specimen date and specimen date of previous positive sample |
| Prosthesis | ‘Y’ if any OPCS procedure code category like “*PROSTHESIS*” in any prior or current inpatient episode |
| Surgical wound | ‘Y’ if any ICD10 diagnosis code in Table S3 in current inpatient episode or prior inpatient episode in same admission spell |
| Urinary catheter | ‘Y’ if any OPCS procedure code category of “URETHRAL CATHETERISATION OF BLADDER” in current inpatient episode or prior inpatient episode in same admission spell |
| Other | - |
| Treatment (e.g. antibiotic given, wound drained, catheter removed) | - |
| Source of bacteraemia & associated infections | - |
| Source of bacteraemia | - |
| Certainty | - |
| Associated clinical infection | - |
| Certainty | - |
| Specialty where infection thought to have been acquired (if inpatient) |  |
| Augmented care specialty | - |
| Treatment specialty | - |
| Date (in specialty) from | - |
| Date (in specialty) to | - |
| Prior healthcare interactions in this trust | Any inpatient episodes or outpatient appointments attended within 12 weeks of specimen date |
| Prior healthcare interactions in other trust | - |
| ***C. difficile:*** |  |
| Best estimate of date of onset of diarrhoea | - |
| Antimicrobial usage |  |
| Was patient on antimicrobials when specimen was taken | - |
| Was patient on any other antimicrobials in preceding 7 days | - |
| Prior healthcare interactions in this trust | Any inpatient episodes or outpatient appointments attended within 12 weeks of specimen date |
| Prior healthcare interactions in other trust | - |
| Specialty where infection thought to have been acquired (if inpatient) |  |
| Augmented care specialty | - |
| Treatment specialty | - |
| Date (in specialty) from | - |
| Date (in specialty) to | - |
| Discharge date | - |
| Discharge type | - |
| Total number of beds (in whole ward or unit) | - |
| Ward type (e.g. single room, 4-bedded bay) | - |
| Reference laboratory result |  |
| Was the specimen sent for typing | - |
| Date sent | - |
| Specimen category | - |
| ***E. coli:*** | **-** |
| Most likely primary focus | - |
| Factors directly predisposing to this episode | - |
| Urinary catheterisation | - |
| Vascular access device | - |
| Other invasive/indwelling device | - |
| Surgical or other invasive procedure | - |
| Neutropenia | - |
| Wound/ulcer | - |
| Other factors | - |
| Is this episode likely to be HCAI | - |
| If yes, where from (e.g. current admission, previous acute admission) | - |
| ***Renal:*** |  |
| Usual provider of renal care: |  |
| Mother unit (hub) | Assumed to be ‘OUH’ |
| Satellite unit | - |
| Other & non-UK etc | - |
| Dialysis details: |  |
| Modality | For any inpatient episodes (including current episode) within 60 days of specimen date:  ‘Haemodialysis’ if any OPCS procedure code label like “HAEMODIALYSIS*”  ‘Haemofiltration’ if any OPCS procedure code label like “HAEMOFILTRATION*”  ‘Peritoneal’ if any OPCS procedure code label like “PERITONEAL*” |
| Type of access being used | For any prior inpatient episodes, the most recent procedure with an OPCS procedure code label like “*ARTERIOVENOUS FISTULA*” or “*ARTERIOVENOUS SHUNT*” or “*VENOUS CATHETER*”:  ‘AVF’ if “*ARTERIOVENOUS FISTULA*”  ‘AVG’ if “*ARTERIOVENOUS SHUNT*”  'Tunnelled venous catheter' if “*TUNNELLED VENOUS CATHETER*”  'Non-tunnelled venous catheter' if “*CENTRAL VENOUS CATHETER*” |
| Catheter last 28/7 | - |
| If Yes, what type | - |
